# Association of statin treatment with hepatocellular carcinoma risk in end-stage kidney disease patients with chronic viral hepatitis

**DOI:** 10.1038/s41598-022-14713-w

**Published:** 2022-06-25

**Authors:** Hyung Woo Kim, Young Su Joo, Shin Chan Kang, Hee Byung Koh, Seung Hyeok Han, Tae-Hyun Yoo, Shin-Wook Kang, Jung Tak Park

**Affiliations:** 1 Department of Internal Medicine, College of Medicine, Institute of Kidney Disease Research, Yonsei University, 50-1 Yonsei-ro, Seodaemun-gu, Seoul, 03722 South Korea; 2 Division of Nephrology, Department of Internal Medicine, Yongin Severance Hospital, Yongin, Gyeonggi-do South Korea; 3 Division of Nephrology, Department of Internal Medicine, Uijeongbu Eulji University Medical Center, Uijeongbu, Gyeonggi-do South Korea

**Keywords:** Gastroenterology, Nephrology

## Abstract

Statin use in end-stage kidney disease (ESKD) patients are not encouraged due to low cardioprotective effects. Although the risk of hepatocellular carcinoma (HCC), a frequently occurring cancer in East Asia, is elevated in ESKD patients, the relationship between statins and HCC is not known despite its possible chemopreventive effect. The relationship between statin use and HCC development in ESKD patients with chronic hepatitis was evaluated. In total, 6165 dialysis patients with chronic hepatitis B or C were selected from a national health insurance database. Patients prescribed with ≥ 28 cumulative defined daily doses of statins during the first 3 months after dialysis commencement were defined as statin users, while those not prescribed with statins were considered as non-users. Primary outcome was the first diagnosis of HCC. Sub-distribution hazard model with inverse probability of treatment weighting was used to estimate HCC risk considering death as competing risk. During a median follow-up of 2.8 years, HCC occurred in 114 (3.2%) statin non-users and 33 (1.2%) statin users. The HCC risk was 41% lower in statin users than in non-users (sub-distribution hazard ratio, 0.59; 95% confidence interval [CI], 0.42–0.81). The weighted incidence rate of HCC was lower in statin users than in statin non-users (incidence rate difference, − 3.7; 95% CI − 5.7 to − 1.7; *P* < 0.001). Incidence rate ratio (IRR) was also consistent with other analyses (IRR, 0.56; 95% CI, 0.41 to 0.78; *P* < 0.001). Statin use was associated with a lower risk of incident HCC in dialysis patients with chronic hepatitis B or C infection.

## Introduction

Although statins, 3-hydroxy-3-methylglutaryl CoA (HMG-CoA) reductase inhibitors, are a mainstay in the primary and secondary prevention of cardiovascular disease in the general population, the benefits of statins in patients undergoing maintenance dialysis are controversial. Large-sized randomized trials have failed to reveal the beneficial effects of statins on cardiovascular outcomes in patients on maintenance dialysis suggesting a relative resistance against statin effect in this population^1–3^. Therefore, current guidelines do not recommend the initiation of statins for patients undergoing dialysis.

The risk of hepatocellular carcinoma (HCC), one of the most frequently occurring cancer types in East Asia^4^, is relatively high in patients undergoing maintenance dialysis. In an evaluation of 92,348 chronic dialysis patients in Taiwan, the standardized incidence ratio for HCC in patients undergoing dialysis was 1.4, suggesting a significant increase in HCC incidence compared to that in the general population^5^. Impaired immunity, deoxyribonucleic acid (DNA) repair, and oxidative stress defense due to the accumulation of uremic toxins and chronic inflammatory status have been suggested as possible reasons for the increased cancer risk in this patient group^6^. In addition, dialysis patients are more prone to chronic hepatitis due to their immunocompromised status and an increased cross-contamination risk^7,8^. Chronic hepatitis B virus (HBV) and hepatitis C virus (HCV) infections are major risk factors for HCC development^9^.

In addition to their cardiovascular protective effects, statins have been recognized for their pleiotropic properties. Recently, statin use has been proposed to have beneficial effects against multiple cancer types^10^. In particular, several observational studies and experimental investigations have revealed the preventive and therapeutic potential of statins against HCC. The inhibition of HMG-CoA reductase by statins has been found to control the production of mevalonate and its downstream metabolites, which play pivotal roles in HCC growth and apoptosis^11^. However, despite the high HCC incidence among dialysis patients, the relationship between statin use and HCC development in this group has not been elucidated.

Therefore, in this study, the association between statin use and the incidence of HCC was examined in patients with chronic HBV or HCV infection in whom dialysis had been newly initiated. This was done by evaluating claims information from a nationwide health insurance database.

## Methods

### Data source

More than 98% of the Korean population is enrolled in a mandatory Korean National Health Insurance Service (NHIS) program, and the remaining people receive government benefits as they are included in the lowest-income bracket. The Health Insurance Review and Assessment Service (HIRA) is a national organization that reviews and evaluates healthcare costs and quality of care. Healthcare providers in Korea are obligated to participate in this program. Information from the HIRA database, including data on patient demographics, prescriptions, treatments, and diagnoses, were reviewed in this study. The present study was carried out in accordance with the Declaration of Helsinki and was approved by the institutional review board of the Yonsei University Health System (4-2019-0501). The requirement for informed consent was waived because of the retrospective nature of the study. De-identification was performed, and data usage was permitted by the national health information data request review committee of the HIRA.

### Study population

**Figure 1:**
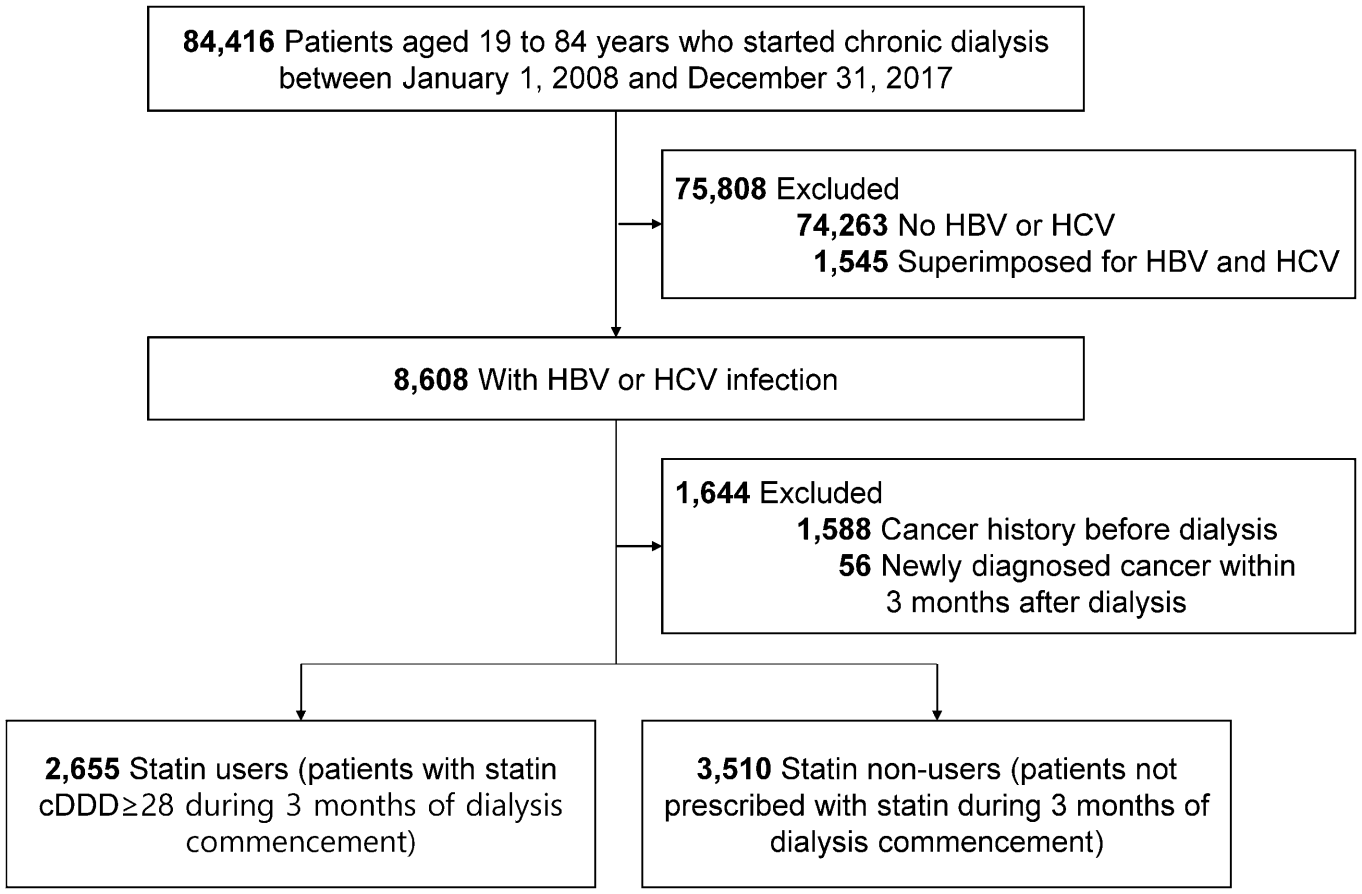
Flow diagram of study inclusion. *HBV* hepatitis B virus, *HCV* hepatitis C virus, *cDDD* cumulative daily defined dose.

Patients who had initiated maintenance dialysis between January 1, 2009, and December 31, 2017, were initially screened. Dialysis patients in Korea are supported by a copayment assistance policy that covers rare, incurable, malignant, or severe and burdensome diseases through the NHIS. Since the recipients of this program are separately coded, dialysis patients were identifiable in the HIRA database. Maintenance dialysis was defined as the administration of dialysis for at least 3 months. Among the screened patients, those aged ≥ 19 and < 85 years and diagnosed with mono-infection with either HBV or HCV were included. Patients with a history of cancer or organ transplantation prior to dialysis initiation were excluded. A total of 6165 patients undergoing maintenance dialysis with chronic viral hepatitis infection were included in the final analysis (Fig. 1).

### Data collection

Baseline demographic data, including data on age and sex, were collected. Comorbidities including diabetes, dyslipidemia, coronary heart disease, congestive heart disease, peripheral vascular disease, cerebral vascular disease, liver cirrhosis, alcoholic liver disease, and fatty liver disease were defined based on the 10th revision of the International Statistical Classification of Diseases and Related Health Problems (ICD-10) codes and claim records. Data on the presence of chronic hepatitis and the type of viral hepatitis infection were also retrieved. Chronic HBV infection was defined using the codes B16.0, B16.1, B16.2, B16.9, B17.0, B18.0, and B18.1. Chronic HCV infection was defined using the codes B17.1 and B18.2. Medication information was acquired using Anatomical Therapeutic Chemical Classification codes. Detailed operational definitions of comorbidities and medications are provided in Supplementary Table S1.

### Statin exposure and outcome measurements

Statin usage was expressed in terms of the defined daily dose (DDD) according to the World Health Organization definition^12^. Exposure duration was determined by cDDD, which was defined as the sum of the DDDs of any statin during the follow-up period. Atorvastatin, simvastatin, pitavastatin, fluvastatin, rosuvastatin, pravastatin, and lovastatin were identified as statins. Patients who received more than or equal to 28 cDDD of statins in the first 3 months after dialysis commencement were defined as statin users. Statin non-users were defined as those who did not receive statin therapy during this period. The primary outcome was the first diagnosis of HCC, which was identified using the ICD-10 C22.0 code. Data on all-cause mortality events were also collected for competing risk analysis. The observation period ended on December 31, 2018.

### Statistical analysis

Continuous variables are presented as the means with standard deviations, and categorical variables are expressed as numbers with percentages. For the main analysis, inverse probability of treatment weighting (IPTW) based on the propensity score was used to balance baseline characteristics between statin users and non-users. Variables included in the propensity score were age; sex; comorbidities of dyslipidemia, diabetes, coronary heart disease, congestive heart failure, peripheral vascular disease, cerebrovascular disease, and liver disease (liver cirrhosis, alcoholic liver disease, and fatty liver disease); and the use of aspirin and antiviral agents. Each observation was weighted by the inverse of the probability of patients receiving a statin, which resulted in a pseudo-population wherein exposure was independent of the measured cofounders^13^. Standardized mean differences were determined to confirm the balance between the groups. Variables were considered to be well balanced when the standardized mean differences after IPTW were < 0.1^14^. To explore the association between statin use and HCC incidence, doubly robust estimation was used, in which the competing risk model was adjusted for covariates included in IPTW estimation^15^. All-cause death before the primary outcome was considered a competing risk^16^. Weighted cumulative incidence curve was presented with weighted incidence rate (IR), incidence rate difference (IRD), and incidence rate ratio (IRR) with 95% confidence intervals (CIs). Weighted IR and IRD were presented as per 1000 person-years. Gray’s test for the equivalence of cumulative incidence function was used to compare the weighted cumulative incidence by group^17^. The sub-distribution hazard ratio (sHR) was assessed with all-cause death as a competing risk to evaluate the association between statin use and HCC incidence. All-cause mortality between the groups was compared using Kaplan–Meier methods. Sensitivity analyses were performed to confirm the main findings.

First, to clarify the potential causal relationship, a dose–response relationship was examined. Patients were categorized as non-users (< 28 cDDD), inconsistent users (< 180 cDDD), and consistent users (≥ 180 cDDD) during the first year after dialysis commencement. Those diagnosed with any type of cancer or who died within 1 year after dialysis initiation were excluded from this analysis. Second, a comparison was performed for patients who had or had not consistently received statin treatment before dialysis initiation. Third, an analysis was performed by excluding patients who had undergone peritoneal dialysis to minimize the effect of the dialysis modality. Fourth, an analysis was performed in patients with and without liver cirrhosis to identify the effect of underlying liver disease on the outcome. Fifth, whether associations with outcomes depended on different types of statins (lipophilic or hydrophilic) were evaluated. Sixth, evaluations were performed without IPTW and without competing risk analysis. Finally, assessments were made in a propensity score-matched cohort in which statin users were matched 1:1 with non-users. All statistical analyses were performed using R (version 3.5.1; http://www.r-project.org; R Foundation for Statistical Computing, Vienna, Austria) and SAS Enterprise Guide (version 6.1; SAS Institute), with *P* values < 0.05 being considered significant.

## Results

### Participants

The baseline characteristics of the enrolled patients are shown in Table 1. The mean age was 60.3 ± 12.7 years, and 37.2% of patients were female. Patients with chronic HBV infection comprised 62.0% of the population, while 38.0% of patients were diagnosed with chronic HCV infection. Among the 6165 patients enrolled, 2655 were statin users, and 3510 were statin non-users. Statin users were more likely to have a history of diabetes, dyslipidemia, coronary heart disease, and vascular disease, whereas severe liver diseases (liver cirrhosis, alcoholic liver disease) were less common. There was no difference in the proportion of fatty liver disease between two groups. After IPTW adjustment, the standardized mean differences showed that all variables were well balanced.

**Table 1:** Baseline characteristics according to statin use.

Variables	Total (N = 6165)	Statin non-user (N = 3510)	Statin user (N = 2655)	Standardized mean difference before IPTW	Standardized mean difference after IPTW
**Type of chronic viral hepatitis**					
HBV	3824 (62.0)	2264 (64.5)	1560 (58.8)	–	–
HCV	2341 (38.0)	1246 (35.5)	1095 (41.2)	–	–
**Demographic data**					
Age, yr	60.3 (12.7)	60.0 (13.0)	60.8 (12.1)	0.068	< 0.001
Female	2294 (37.2)	1255 (35.8)	1039 (39.1)	0.070	0.004
**Comorbidities**					
Diabetes	4517 (73.3)	2319 (66.1)	2198 (82.8)	0.390	0.014
Dyslipidemia	1647 (26.7)	589 (16.8)	1058 (39.8)	0.530	0.006
Coronary heart disease	522 (8.5)	164 (4.7)	358 (13.5)	0.310	0.004
Congestive heart failure	899 (14.6)	462 (13.2)	437 (46.5)	0.093	0.003
Peripheral vascular disease	609 (9.9)	298 (8.5)	311 (11.7)	0.107	0.001
Cerebral vascular disease	896 (14.5)	440 (12.5)	456 (17.2)	0.131	0.017
Liver cirrhosis	566 (9.2)	435 (12.4)	131 (4.9)	0.268	0.008
Alcoholic liver disease	182 (3.0)	150 (4.3)	32 (1.2)	0.189	0.010
Fatty liver disease	344 (5.6)	188 (5.4)	156 (5.9)	0.023	0.001
**Medications**					
Aspirin	2216 (35.9)	936 (26.7)	1280 (48.2)	0.457	0.007
Antiviral agents	811 (13.2)	530 (15.1)	281 (10.6)	0.135	0.003
**Peritoneal dialysis**					
Ever experienced	1061 (17.2)	579 (16.5)	482 (18.2)	–	–

All continuous variables are expressed as means and standard deviations. All categorical variables are expressed as numbers and percentages. Comorbidities were based on the claims data within 1 year before chronic dialysis commencement.

*IPTW* inverse probability of treatment weighting, *HBV* hepatitis B virus, *HCV* hepatitis C virus.

### Incidence of hepatocellular carcinoma

**Figure 2:**
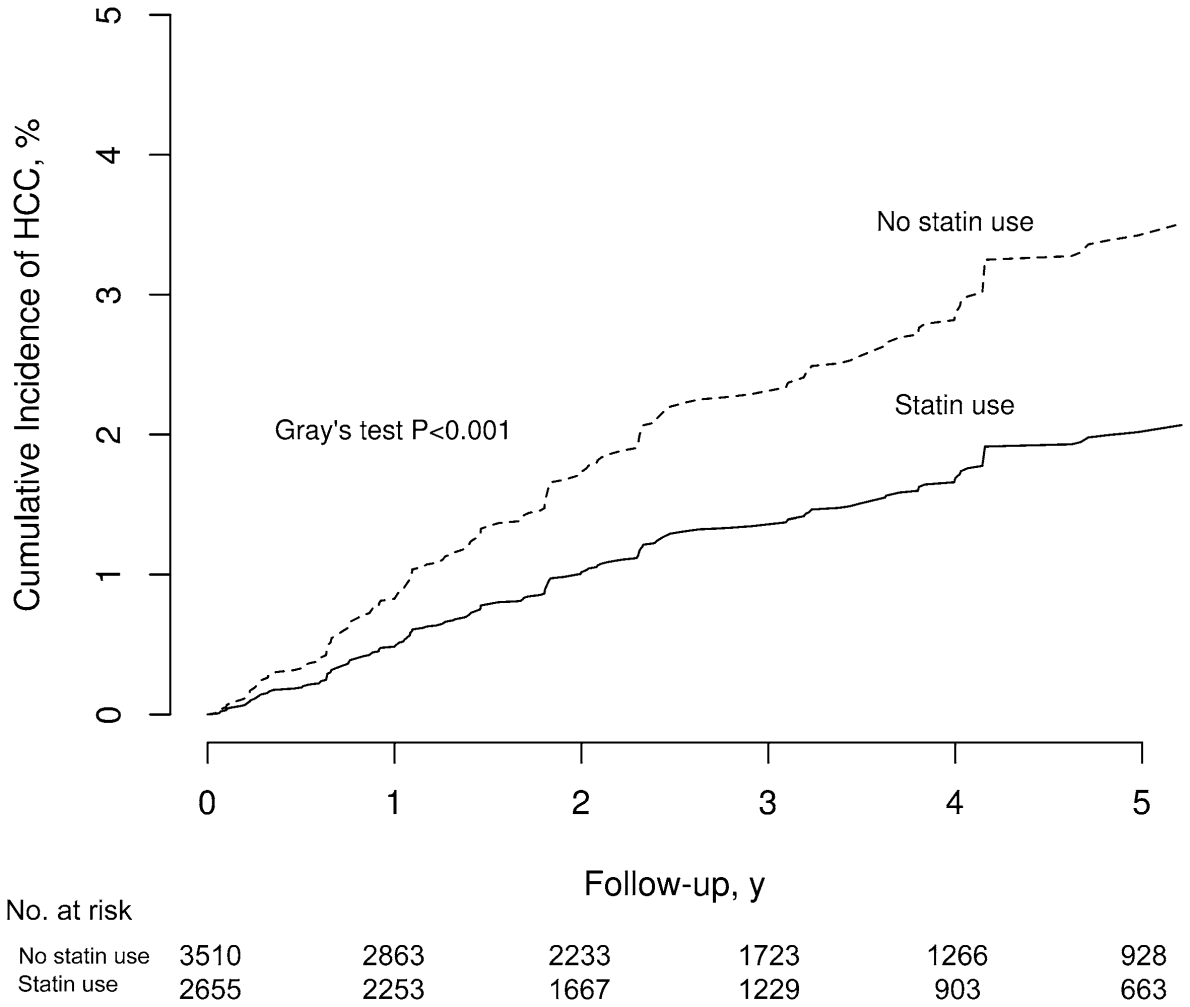
Weighted cumulative incidence curve of hepatocellular carcinoma in dialysis patients with chronic viral hepatitis according to statin use.

During a median follow-up of 2.8 years (interquartile range, 1.4–3.4 years), incident HCC occurred in 147 (2.4%) patients, while all-cause death was observed in 1894 (30.7%) patients. Incident HCC was seen in 114 (3.2%) statin non-users and 33 (1.2%) statin users. The weighted IR of HCC was lower in statin users than in statin non-users (IRD, − 3.7; 95% CI, − 5.7 to − 1.7; *P* < 0.001). Incidence rate ratio (IRR) suggested that statin user was associated with a lower risk for HCC than statin non-user (IRR, 0.56; 95% CI, 0.41–0.78; *P* < 0.001). The cumulative incidence was significantly higher in statin non-users than in statin users (Gray’s test, *P* < 0.001, Fig. 2). When the sub-distribution hazard ratio of HCC was assessed, the risk for incident HCC was 41% lower in statin users than in statin non-users (sHR, 0.59; 95% CI, 0.42–0.81;* P* = 0.001). This relationship remained significant even after adjustments were made for confounding factors (Table 2, Supplementary Table S2). In the weighted Kaplan–Meier analysis, statin use was associated with the lower risk of overall survival (log-rank test *P* < 0.001) (Supplementary Fig. S1).

**Table 2:** Association between statin use and the risk of incident hepatocellular carcinoma in dialysis patients with chronic viral hepatitis.

Treatment group	Event no./Total no.	IR (95% CI)^a^	IRD (95% CI)^a^	IRR (95% CI)^a^	Sub-distribution hazard ratio (95% CI)^a,b^			
					Unadjusted	*P* value	Adjusted^c^	*P* value
No statin use	114/3510	8.4 (7.0 to 10.1)	0 (reference)	1.00 (reference)	1.00 (reference)	–	1.00 (reference)	–
Statin use	33/2655	4.7 (3.6 to 6.2)	− 3.7 (− 5.7 to − 1.7)	0.56 (0.41 to 0.78)	0.59 (0.42 to 0.81)	0.001	0.55 (0.39 to 0.77)	< 0.001

^a^Estimated from the inverse probability of treatment weighted cohort.

^b^All-cause death was considered as a competing risk.

^c^Multivariable models were adjusted for age, sex, dyslipidemia, diabetes, coronary heart disease, congestive heart failure, peripheral vascular disease, cerebrovascular disease, liver disease (liver cirrhosis, alcoholic liver disease, and fatty liver disease), aspirin use, and antiviral agent use.

Incidence rate and incidence rate difference were presented as per 1000 person-years.

*IR* incidence rate, *IRD* incidence rate difference, *IRR* incidence rate ratio, *CI* confidence interval.

### Incidence of hepatocellular carcinoma according to the type of viral hepatitis

**Figure 3:**
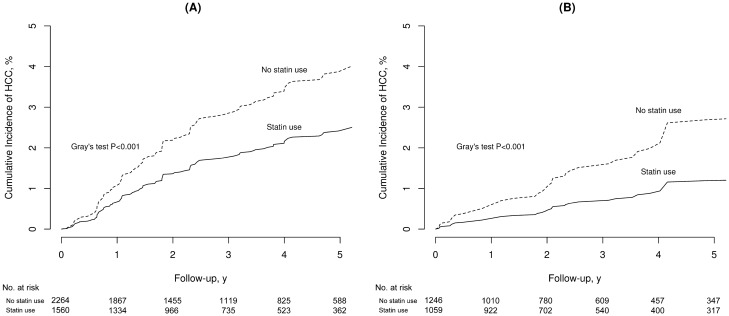
Weighted cumulative incidence curves of hepatocellular carcinoma in dialysis patients with chronic hepatitis B (**A**) and hepatitis C (**B**) according to statin use.

The incidence of HCC among statin users and non-users was also evaluated separately according to viral hepatitis type. Among those with chronic HBV infection, the weighted IR of HCC was lower in statin users than in statin non-users (IRD, − 4.0; 95% CI, − 6.8 to − 1.2; *P* = 0.006). IRR suggested that statin user was associated with a lower risk for HCC than statin non-user (IRR, 0.59; 95% CI, 0.40–0.88; *P* = 0.008). The cumulative incidence was significantly higher in statin non-users than in statin users (Gray’s test, *P* < 0.001, Fig. 3A). In addition, the risk for incident HCC was significantly lower in statin users than in statin non-users (sHR, 0.62; 95% CI, 0.42–0.91; *P* = 0.015). A similar finding was observed among patients with chronic HCV infection (Fig. 3B and Table 3).

**Table 3:** Association between statin use and the risk of incident hepatocellular carcinoma in dialysis patients according to viral hepatitis type.

Treatment group	Event no./Total no.	IR (95% CI)^a^	IRD (95% CI)^a^	IRR (95% CI)^a^	Sub-distribution hazard ratio (95% CI)^a,b^			
					Unadjusted	*P* value	Adjusted^c^	*P* value
**HBV cohort**								
No statin use	84/2264	9.8 (8.0 to 12.2)	0 (reference)	1.00 (reference)	1.00 (reference)	–	1.00 (reference)	–
Statin use	25/1560	5.8 (4.2 to 8.1)	− 4.0 (− 6.8 to − 1.2)	0.59 (0.40 to 0.88)	0.62 (0.42 to 0.91)	0.015	0.58 (0.39 to 0.86)	0.007
**HCV cohort**								
No statin use	30/1246	6.3 (4.4 to 8.9)	0 (reference)	1.00 (reference)	1.00 (reference)	–	1.00 (reference)	–
Statin use	8/1095	2.7 (1.5 to 4.7)	− 3.6 (− 6.3 to − 0.9)	0.43 (0.22 to 0.83)	0.44 (0.23 to 0.85)	0.015	0.39 (0.20 to 0.74)	0.004

^a^Estimated from the inverse probability of treatment weighted cohort.

^b^All-cause death was considered as a competing risk.

^c^Multivariable models were adjusted for age, sex, dyslipidemia, diabetes, coronary heart disease, congestive heart failure, peripheral vascular disease, cerebrovascular disease, liver disease (liver cirrhosis, alcoholic liver disease, and fatty liver disease), aspirin use, and antiviral agent use.

Incidence rate and incidence rate difference were presented as per 1000 person-years.

*IR* incidence rate, *IRD* incidence rate difference, *IRR* incidence rate ratio, *CI* confidence interval.

### Sensitivity analyses

When patients were re-classified as non-users (< 28 cumulative defined daily dose [cDDD]), inconsistent users (≥ 28, < 180 cDDD), and consistent users (≥ 180 cDDD), consistent users were associated with a lower risk of incident HCC (adjusted sHR, 0.51; 95% CI, 0.31–0.83; *P* = 0.007) than non-users. However, such a relationship was not found with inconsistent users (adjusted sHR, 0.95; 95% CI, 0.60–1.50; *P* = 0.823; Supplementary Table S3). When the effect of consistent statin use was considered, consistent statin use after dialysis commencement was related to a decreased risk of HCC development (Supplementary Table S4). In addition, evaluation of hemodialysis patients revealed findings similar to the main results, suggesting that the relationship between statin use and HCC incidence is independent of the dialysis modality (adjusted sHR, 0.53; 95% CI, 0.37–0.77; *P* < 0.001; Supplementary Table S4). However, in an evaluation based on the presence of liver cirrhosis, the lower risk of incident HCC in statin users was lost among those with underlying liver cirrhosis (adjusted sHR, 0.82; 95% CI, 0.50 to 1.35; *P* = 0.434; Supplementary Table S5). When considering the type of statin, incident HCC risk was significantly reduced in both lipophilic (adjusted sHR, 0.58; 95% CI, 0.41–0.82; P = 0.004) and hydrophilic statin (adjusted sHR, 0.47; 95% CI, 0.27–0.82; P = 0.015) users compared to non-users (Supplementary Table S6). Assessments performed without IPTW (adjusted sHR, 0.50; 95% CI, 0.33–0.77;* P* = 0.001) or without considering the competing risk (adjusted HR, 0.52; 95% CI, 0.37–0.72;* P* < 0.001) also revealed that the incident HCC risk was lower in statin users (Supplementary Tables S7and S8). Lastly, additional assessments using a 1:1 propensity score matched cohort were consistent with the main analysis (adjusted sHR 0.42; 95% CI, 0.27–0.68; P = < 0.001) (Supplementary Tables S9 and S10).

## Discussion

Statin use was associated with a reduced risk of HCC incidence in a nationwide evaluation of 6165 dialysis patients with chronic viral hepatitis, a high-risk group for HCC. During a median follow-up duration of 2.8 years after dialysis commencement, the risk of HCC was 41% lower in statin users than in statin non-users. This relationship was independent of viral hepatitis type, comorbidities, or the prescription of concomitant medications known to influence HCC risk, such as aspirin and antiviral agents.

The reduction in incident HCC risk found in this study has been previously recognized in several patient populations. Statin use was associated with a decreased risk of HCC and HCC-related death in high-risk groups such as chronic HBV and HCV carriers^18–24^, patients with non-alcoholic liver disease^25^, or diabetes mellitus^26,27^. In a recent nested case–control study, statin use was demonstrated to lower the risk of HCC development in the general population^28^. CKD patients aged over 50 who have not yet initiated dialysis are recommended to use statin for cardiovascular disease prevention in current guideline. Considering the high frequency of risk factors for HCC in CKD patients, statin use might have a protective effect on HCC in patients with CKD regardless of viral hepatitis infection. Meanwhile, dialysis patients have been considered “statin resistant” based on clinical studies that have failed to recapture the efficacy of statins against cardiovascular disease noticed among the non-dialysis population. In large-scale prospective trials, such as the Die Deutsche Diabetes Dialyse (4D) study, the rate of mortality from all cardiac causes did not differ between the statin and placebo groups, despite the significant decline in low-density lipoprotein levels in the statin group^3^. Complex lipid abnormalities such as highly oxidized or carbamylated lipoproteins found in uremic patients have been suspected as a cause of this discrepancy in the statin effect between patients undergoing and not undergoing dialysis^29^. In addition, the increase in intracellular cholesterol synthesis, which is not fully inhibited by statin use, under chronic inflammatory conditions such as chronic dialysis has also been postulated to play a role in statin resistance^30^. The fact that the risk of HCC development was significantly lower among statin users in this study suggests that patients undergoing dialysis are not resistant to the antineoplastic effect of statins found in those without kidney disease. Considering that the risk of HCC development is increased among dialysis patients compared to that in the general population and that the initiation of statin therapy is discouraged by current guidelines, the results of the current study may open the possibility of statin use being suggested in dialysis patients with high HCC risk.

Statin use was not associated with HCC risk reduction in the subgroups with underlying liver cirrhosis. In contrast to this finding, several previous evaluations in the non-dialysis population have shown that the preventive effect of statins against HCC was maintained in patients with liver cirrhosis. A recent nested case–control study of 1642 HCC patients revealed that statin use was significantly associated with a reduction in HCC incidence in patients with liver cirrhosis as well as in those without liver cirrhosis^28^. The relatively small number of statin users with liver cirrhosis in this study could have played a role in producing a statistically insignificant relationship between statin use and HCC incidence. However, the probability that statin treatment alone may not be sufficient to prevent HCC development in high-risk patients, such as those with liver cirrhosis under uremic conditions, should also be considered.

In this study, only 39.8% of statin users had dyslipidemia. Since patients with kidney disease are a high-risk group for cardiovascular diseases, statins would have been also prescribed for cardio-protective purposes, in addition to the goal of managing dyslipidemia. The fact that 37.1% of the participants were reported to have a medical history of cardiovascular disease (coronary heart disease, congestive heart failure, peripheral vascular disease, or cerebral vascular disease) in this study supports this possibility.

The preventive effects of statins on HCC development could be attributed to several mechanisms. Simvastatin, fluvastatin, and lovastatin have been shown to induce a selective apoptotic effect in human HCC cell lines^31,32^. Statins, including atorvastatin, have been found to block MYC phosphorylation, resulting in tumor-suppressive effects^33,34^. Among patients with HBV, the transcriptional activation of HBV protein X alters the expression of growth control genes such as *Ras*, *Raf*, *MAPK*, and *ERK*^35^. By inhibiting the mevalonate pathway, statins have been demonstrated to effectively prevent the detrimental consequences of the signaling proteins encoded by these genes^11^. HCV is known to stimulate nuclear factor κB, resulting in chronic inflammation, a neoplastic-prone state^36^. HCV infection also promotes cell growth by downregulating members of the growth arrest and DNA damage (Gadd45) gene family^37^. Statins have been recognized to effectively counter these effects, which could lead to anti-tumor properties.

This study has several strengths. First, this study evaluated an unbiased selection from a nationwide dialysis population. Due to the obligatory copayment assistance policy of the Korean NHIS, dialysis patients and those diagnosed with cancerous disease are coded separately in the HIRA database. This allowed the detection of an entire population of patients undergoing chronic dialysis as well as those diagnosed with HCC during the study period. Second, double robust estimation, adjusting for covariates after IPTW application, was used. Since statin treatment is dependent on underlying metabolic abnormalities and comorbidities that may affect HCC development, such an evaluation strategy would further strengthen the possible independent association between statin use and incident HCC.

However, the findings of this study should be interpreted in light of the following limitations. First, limitations due to the observational nature of the study should be considered. Although a significant association between statin use and HCC development was found, the cause-effect relationship should be further assessed in future prospective evaluations. Second, due to the nature of health insurance claim data, the possibility of missing diagnostic codes for comorbidities including HBV or HCV infection, diabetes, dyslipidemia, and liver disease cannot be excluded. While claims database analysis has advantage of utilizing a large sized data, this analysis is potentially susceptible to errors from inaccuracies. However, screening for viral hepatitis (both B and C) is routinely performed before starting dialysis in real-world practice, and HIRA periodically performs a nationwide obligatory quality assessment which includes viral serologic tests, lowering the possibility of HBV or HCV carriers not being detected. In addition, specific malignant disease insurance code that were issued by the Korea NHIS (V193) was utilized for accurate HCC diagnosis although tumor staging data were not available. Third, as the evaluation used claims data from a national insurance service database, potential confounding variables including lifestyle factors; anthropometric factors; and laboratory information including hepatitis viral copy number, liver function, tumor markers, and lipid abnormalities could not be examined. Further analyses considering laboratory information associated with liver function would be needed to reduce the possibility of selection bias. Fourth, this study was conducted in a single nation, with a predominately Asian population, in which HBV and HCV infection rates are relatively higher than those in Western countries. Therefore, to generalize these findings, assessments including other populations wherein viral hepatitis is not the predominant cause of HCC should be performed.

In conclusion, this nationwide observational study showed that statin use was associated with a reduced risk of incident HCC in chronic dialysis patients with HBV or HCV infection. The decrease in risk was independent of comorbidities and was evident regardless of the viral hepatitis type. Further prospective trials are needed to verify the protective effects of statins against HCC in this patient group.

## Supplementary Information

Supplementary Information.

## Data Availability

The data underlying this article are available through the Health Insurance Review and Assessment Service (HIRA) (https://opendata.hira.or.kr). Data usage was permitted by the national health information data request review committee of the HIRA.
