# Supplementary material for: Association of statin treatment with hepatocellular carcinoma risk in end-stage kidney disease patients with chronic viral hepatitis
Source: Sci Rep. 2022 Jun 25;12:10807. doi: 10.1038/s41598-022-14713-w (PMC9233705; doi:10.1038/s41598-022-14713-w)

**Supplementary Material**

Association of statin treatment with hepatocellular carcinoma risk

in end-stage kidney disease patients with chronic viral hepatitis

Hyung Woo Kim^1^, Young Su Joo^1,2^, Shin Chan Kang^3^, Hee Byung Koh^1^, Seung Hyeok Han^1^, Tae-Hyun Yoo^1^, Shin-Wook Kang^1^, and Jung Tak Park^1^

^1^Department of Internal Medicine, College of Medicine, Institute of Kidney Disease Research, Yonsei University, Seoul, South Korea

^2^Division of Nephrology, Department of Internal Medicine, Yongin Severance Hospital, Yongin, Gyeonggi-do, South Korea

^3^Division of Nephrology, Department of Internal Medicine, Uijeongbu Eulji University Medical Center, Uijeongbu, Gyeonggi-do, South Korea

*** Correspondence:**Jung Tak Park
jtpark@yuhs.ac

***Supplementary Table S1.*** Definition of measurements

|  | **Definition** |
| --- | --- |
| **Type of chronic viral hepatitis** |  |
| HBV infection | ICD-10 code: B16.0, B16.1, B16.2, B16.9, B17.0, B18.0, B18.1 |
| HCV infection | ICD-10 code: B17.1, B18.2 |
| **Medications** |  |
| Statins | Medication ATC codes C10 |
| Aspirin | Medication ATC codes B01AC06 |
| Antiviral agent | Medication ATC codes J05AF, J05AP, L03AB |
| **Comorbidities** |  |
| Hepatocellular carcinoma | ICD-10 code: C22.0 |
| Diabetes | ICD-10 code: E10, E11, E12, E13, E14 |
| Dyslipidemia | ICD-10 code: E78.0, E78.1, E78.2, E78.3, E78.4, E78.5 |
| Coronary heart disease | ICD-10 code: I21, I22, I23, I24, I25 |
| Congestive heart failure | ICD-10 code: I43, I50, I09.9, I11.0, I13.0, I13.2, I25.5, I42.0, I42.5, I42.6, I42.7, I42.8, I42.9, P29.0 |
| Cerebrovascular disease | ICD-10 code: G45, G46, I60 ~ I69, H34.0 |
| Peripheral vascular disease | ICD-10 code: I7.0, I7.1, I73.1, I73.8, I73.9, I77.1, I79.0, I79.2, K55.1, K55.8, K55.9, Z958, Z95.9 |
| Liver cirrhosis | ICD-10 code: K74 |
| Alcoholic liver disease | ICD-10 code: K70 |
| Fatty liver disease | ICD-10 code: K70.0, K75.8, K76.0 |

***Abbreviations:*** HBV, hepatitis B virus; HCV, hepatitis C virus; ICD, International Statistical Classification of Diseases and Related Health Problems; ATC, Anatomical Therapeutic Chemical.

***Supplementary Table S2****.* The risk of incident hepatocellular carcinoma in dialysis patients with chronic viral hepatitis

| **Treatment**  **Group** | **Sub-distribution Hazard Ratio (95% CI)^a,b^** | | | |
| --- | --- | --- | --- | --- |
|  | Unadjusted | *P* value | Adjusted^c^ | *P* value |
| No statin use | 1.00 (reference) | - | 1.00 (reference) | - |
| Statin use | 0.59 (0.42 to 0.81) | 0.001 | 0.55 (0.39 to 0.77) | <0.001 |
| Age | 1.00 (0.99 to 1.01) | 0.912 | 1.02 (1.01 to 1.04) | 0.005 |
| Female | 0.40 (0.27 to 0.59) | <0.001 | 0.50 (0.33 to 0.76) | 0.001 |
| **Comorbidities** |  |  |  |  |
| Diabetes | 1.48 (1.01 to 2.17) | 0.045 | 1.50 (1.03 to 2.20) | 0.037 |
| Dyslipidemia | 0.82 (0.57 to 1.17) | 0.279 | 0.90 (0.61 to 1.33) | 0.596 |
| Coronary heart disease | 1.06 (0.63 to 1.79) | 0.049 | 1.13 (0.66 to 1.91) | 0.664 |
| Congestive heart failure | 1.34 (0.90 to 1.98) | 0.146 | 1.55 (1.03 to 2.33) | 0.038 |
| Peripheral vascular disease | 1.08 (0.67 to 1.74) | 0.762 | 1.26 (0.77 to 2.07) | 0.350 |
| Cerebral vascular disease | 0.69 (0.43 to 1.13) | 0.140 | 0.81 (0.48 to 1.35) | 0.412 |
| Liver cirrhosis | 10.00 (7.38 to 13.56) | <0.001 | 5.37 (3.68 to 7.86) | <0.001 |
| Alcoholic liver disease | 4.38 (2.76 to 6.94) | <0.001 | 1.87 (1.11 to 3.14) | 0.019 |
| Fatty liver disease | 1.77 (1.05 to 3.00) | 0.033 | 1.53 (0.89 to 2.62) | 0.126 |
| **Medications** |  |  |  |  |
| Aspirin use | 0.82 (0.60 to 1.14) | 0.241 | 1.08 (0.77 to 1.51) | 0.668 |
| Antiviral agents use | 6.36 (4.70 to 8.63) | <0.001 | 3.75 (2.55 to 5.51) | <0.001 |

***Note:*** ^a^Estimated from the inverse probability of treatment weighted cohort according to statin use.

^b^All-cause death was considered as a competing risk.

^c^Multivariable models were adjusted for age, sex, dyslipidemia, diabetes, coronary heart disease, congestive heart failure, peripheral vascular disease, cerebrovascular disease, liver disease (liver cirrhosis, alcoholic liver disease, and fatty liver disease), aspirin use, and antiviral agent use.

***Abbreviations:*** CI, confidence interval.

***Supplementary Table S3.*** Association between statin use and the risk of incident hepatocellular carcinoma in dialysis patients with chronic viral hepatitis according to statin use consistency

|  | **Event No./**  **Total No.** | **Sub-distribution Hazard Ratio (95% CI)^a,b,c^** | | | |
| --- | --- | --- | --- | --- | --- |
|  |  | Unadjusted | *P* Value | Adjusted | *P* Value |
| Nonuser (<28 cDDD) | 75/2633 | 1.00 (Reference) | - | 1.00 (Reference) | - |
| Inconsistent user (<180 cDDD) | 17/928 | 0.93 (0.59-1.45) | 0.735 | 0.95 (0.60-1.50) | 0.823 |
| Consistent user (≥180 cDDD) | 12/1543 | 0.52 (0.32-0.84) | 0.007 | 0.51 (0.31-0.83) | 0.007 |

***Note:*** ^a^Estimated from the inverse probability of treatment weighted cohort

^b^All-cause death was considered as a competing risk.

^c^Follow-up began 1 year after dialysis start.

Excluding those diagnosed with any type of cancer or who died within 1 year after dialysis initiation, 5,104 patients were included in this analysis.

***Abbreviations:*** cDDD, cumulative defined daily dose; CI, confidence interval.

***Supplementary Table S4****.* Association between statin use and the risk of incident hepatocellular carcinoma in dialysis patients with chronic viral hepatitis

| **Sensitivity Analysis** | **Event No./**  **Total No.** | **Sub-distribution Hazard Ratio (95% CI) ^a,b^** | | | |
| --- | --- | --- | --- | --- | --- |
|  |  | Unadjusted | *P* Value | Adjusted | *P* Value |
| Among hemodialysis patients only,  Statin use after dialysis vs. No statin use after dialysis | 125/5104 | 0.56 (0.39-0.81) | 0.002 | 0.53 (0.37-0.77) | <0.001 |
| Consistent statin usage before and after dialysis vs. No statin use before and after dialysis | 126/5270 | 0.68 (0.48-0.95) | 0.023 | 0.64 (0.45-0.90) | 0.011 |
| Among patients with statin use before dialysis,  Consistent statin use before and after dialysis vs. Stop statin use after dialysis commencement | 42/2597 | 0.51 (0.26-1.00) | 0.050 | 0.43 (0.22-0.83) | 0.012 |

***Note:*** ^a^Estimated from the inverse probability of treatment weighted cohort

^b^All-cause death was considered as a competing risk.

***Abbreviations:*** CI, confidence interval.

***Supplementary Table S5****.* Association between statin use and the risk of incident hepatocellular carcinoma in dialysis patients with chronic viral hepatitis according to concomitant liver cirrhosis.

| **Cohort** | **Event No./**  **Total No.** | **Sub-distribution Hazard Ratio (95% CI) ^a,b^** | | | |
| --- | --- | --- | --- | --- | --- |
|  |  | Unadjusted | *P* Value | Adjusted | *P* Value |
| Without liver cirrhosis | 80/5599 | 0.31 (0.18-0.52) | <0.001 | 0.31 (0.18-0.53) | <0.001 |
| With liver cirrhosis | 56/566 | 0.77 (0.48-1.23) | 0.275 | 0.82 (0.50-1.35) | 0.434 |

***Note:*** ^a^Estimated from the inverse probability of treatment weighted cohort

^b^All-cause death was considered as a competing risk.

***Abbreviations:*** cDDD, cumulative defined daily dose; CI, confidence interval.

***Supplementary Table S6.*** Association between statin use and the risk of incident hepatocellular carcinoma in dialysis patients with chronic viral hepatitis according to the types of statins

|  | **Event No./**  **Total No.** | **Sub-distribution Hazard Ratio (95% CI)^a,b^** | | | |
| --- | --- | --- | --- | --- | --- |
|  |  | Unadjusted | *P* Value^c^ | Adjusted | *P* Value^c^ |
| Non-user | 114/3510 | 1.00 (Reference) | - | 1.00 (Reference) | - |
| Statin use (lipophilic statin)^d^ | 26/1976 | 0.63 (0.44-0.89) | 0.017 | 0.58 (0.41-0.82) | 0.004 |
| Statin use (hydrophilic statin)^d^ | 7/679 | 0.45 (0.26-0.79) | 0.010 | 0.47 (0.27-0.82) | 0.015 |

***Note:*** ^a^Estimated from the inverse probability of treatment weighted cohort

^b^All-cause death was considered as a competing risk.

^c^Corrected using Bonferroni’s method due to multiple testing

^d^Lipophilic statin included atorvastatin (n=1440), simvastatin (n=286), pitavastatin (n=132), lovastatin (n=4) and fluvastatin (n=114). Hydrophilic statin included rosuvastain (n=515) and pravastatin (n=164).

***Abbreviations:*** CI, confidence interval; HCC, hepatocellular carcinoma.

***Supplementary Table S7.*** Association between statin use and the risk of incident hepatocellular carcinoma in dialysis patients with chronic viral hepatitis, without IPTW analysis

| **Cohort** | **Event No./**  **Total No.** | **Sub-distribution Hazard Ratio (95% CI)^a^** | | | |
| --- | --- | --- | --- | --- | --- |
|  |  | Unadjusted | *P* Value | Adjusted | *P* Value |
| Overall cohort | 147/6165 | 0.40 (0.27-0.59) | <0.001 | 0.50 (0.33-0.77) | 0.001 |
| HBV cohort | 109/3824 | 0.46 (0.30-0.72) | <0.001 | 0.59 (0.36-0.96) | 0.040 |
| HCV cohort | 38/2341 | 0.31 (0.14-0.67) | 0.003 | 0.35 (0.16-0.79) | 0.010 |

***Note:*** ^a^All-cause death was considered as a competing risk.

***Abbreviations:*** IPTW, inverse probability of treatment weighting; HBV, hepatitis B virus; HCV, hepatitis C virus; CI, confidence interval.

***Supplementary Table S8****.* Association between statin use and the risk of incident hepatocellular carcinoma in dialysis patients with chronic viral hepatitis, without considering competing risk

| **Cohort** | **Event No./**  **Total No.** | **Sub-distribution Hazard Ratio (95% CI)^a^** | | | |
| --- | --- | --- | --- | --- | --- |
|  |  | Unadjusted | *P* Value | Adjusted | *P* Value |
| Overall cohort | 147/6165 | 0.56 (0.40-0.78) | <0.001 | 0.52 (0.37-0.72) | <0.001 |
| HBV cohort | 109/3824 | 0.59 (0.40-0.86) | 0.007 | 0.56 (0.37-0.83) | 0.004 |
| HCV cohort | 38/2341 | 0.43 (0.22-0.83) | 0.012 | 0.37 (0.19-0.71) | 0.003 |

***Note:*** ^a^Estimated from the inverse probability of treatment weighted cohort

***Abbreviations:*** cDDD, cumulative defined daily dose; CI, confidence interval.

***Supplementary Table S9.*** Baseline characteristics according to statin use. Statin users were matched 1:1 with non-users by means of propensity scores.

| **Variables** | **Before matching** | | | | | **After matching** | | | | |
| --- | --- | --- | --- | --- | --- | --- | --- | --- | --- | --- |
|  | **Non-user**  **(N=3,510)** | **Statin user**  **(N=2,655)** | ***P* value** | **Standardized difference** |  | | **Non-user**  **(N=2,095)** | **Statin user**  **(N=2,095)** | ***P* value** | **Standardized difference** |
| **Type of chronic viral hepatitis** |  |  |  |  |  | |  |  |  |  |
| HBV | 2264 (64.5) | 1560 (58.8) | <0.001 | - |  | | 1310 (62.5) | 853 (40.7) | 0.034 | - |
| HCV | 1246 (35.5) | 1095 (41.2) | <0.001 | - |  | | 785 (37.5) | 853 (40.7) | 0.034 | - |
| **Demographic data** |  |  |  |  |  | |  |  |  |  |
| Age, yr | 60.0 (13.0) | .60.8 (12.1) | 0.008 | 0.0705 |  | | 60.5 (13.1) | 60.4 (12.3) | 0.848 | -0.0062 |
| Female | 1255 (35.8) | 1039 (39.1) | 0.007 | -0.0692 |  | | 783 (37.4) | 835 (39.9) | 0.106 | -0.0508 |
| **Comorbidities** |  |  |  |  |  | |  |  |  |  |
| Diabetes | 2319 (66.1) | 2198 (82.8) | <0.001 | 0.4428 |  | | 1664 (79.4) | 1663 (79.4) | 1.000 | 0.0002 |
| Dyslipidemia | 589 (16.8) | 1058 (39.8) | <0.001 | 0.4711 |  | | 558 (26.6) | 621 (29.6) | 0.033 | 0.0614 |
| Coronary heart disease | 164 (4.7) | 358 (13.5) | <0.001 | 0.2579 |  | | 152 (7.3) | 161 (7.7) | 0.638 | 0.0126 |
| Congestive heart failure | 462 (13.2) | 437 (46.5) | <0.001 | 0.0889 |  | | 310 (14.8) | 317 (15.1) | 0.795 | 0.0090 |
| Peripheral vascular disease | 298 (8.5) | 311 (11.7) | <0.001 | 0.1002 |  | | 209 (10.0) | 229 (10.9) | 0.337 | 0.0297 |
| Cerebral vascular disease | 440 (12.5) | 456 (17.2) | <0.001 | 0.1230 |  | | 327 (15.6) | 357 (17.0) | 0.225 | 0.0380 |
| Liver cirrhosis | 435 (12.4) | 131 (4.9) | <0.001 | -0.3443 |  | | 114 (5.4) | 126 (6.0) | 0.465 | 0.0264 |
| Alcoholic liver disease | 150 (4.3) | 32 (1.2) | <0.001 | -0.2811 |  | | 29 (1.4) | 32 (1.5) | 0.796 | 0.0131 |
| Fatty liver disease | 188 (5.4) | 156 (5.9) | 0.4010 | 0.0221 |  | | 117 (5.6) | 111 (5.3) | 0.733 | -0.0122 |
| **Medications** |  |  |  |  |  | |  |  |  |  |
| Aspirin | 936 (26.7) | 1280 (48.2) | <0.001 | 0.4311 |  | | 827 (39.5) | 844 (40.3) | 0.614 | 0.0162 |
| Antiviral agents | 530 (15.1) | 281 (10.6) | <0.001 | -0.1468 |  | | 239 (11.4) | 235 (11.2) | 0.884 | -0.0062 |

***Note:*** All continuous variables are expressed as means and standard deviations. All categorical variables are expressed as numbers and percentages. Comorbidities were based on the claims data within 1 year before chronic dialysis commencement.

***Abbreviations:*** HBV, hepatitis B virus; HCV, hepatitis C virus.

***Supplementary Table S10****.* Association between statin use and the risk of incident hepatocellular carcinoma in dialysis patients with chronic viral hepatitis. Statin users were matched 1:1 with non-users by means of propensity scores.

| **Event and Treatment Group^a^** | **Event No./**  **Total No.** | **Sub-distribution Hazard Ratio (95% CI)^a^** | | | |
| --- | --- | --- | --- | --- | --- |
|  |  | Unadjusted | *P* value | Adjusted | *P* value |
| No statin use | 57/2095 | 1.00 (reference) | - | 1.00 (reference) | - |
| Statin use | 26/2095 | 0.48 (0.30-0.77) | 0.002 | 0.42 (0.27-0.68) | <0.001 |

***Note:*** ^a^All-cause death was considered as a competing risk.

***Abbreviations:*** CI, confidence interval.

**Supplemental Figure S1.** Weighted Kaplan-Meier analysis with log-rank test about overall survival in dialysis patients with chronic viral hepatitis according to statin use.


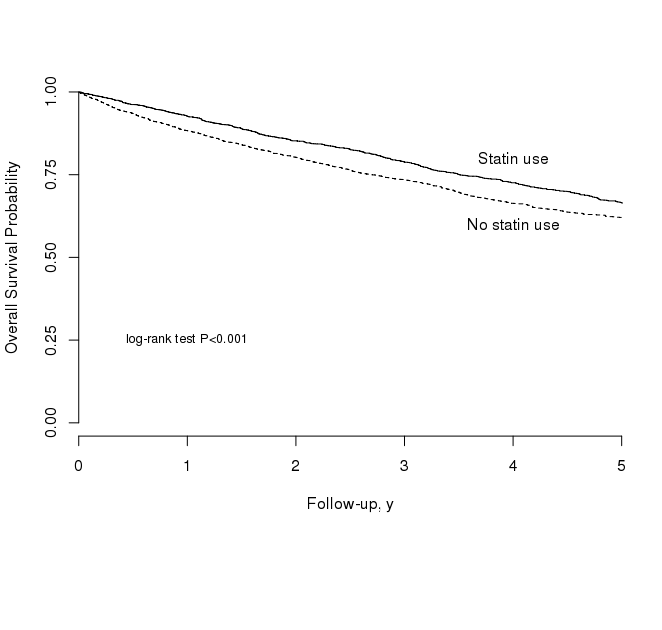

Supplement: Supplementary file 1 — Supplementary Information. [file 41598_2022_14713_MOESM1_ESM.docx]
